# Mycotoxins, Pesticide Residues, and Heavy Metals Analysis of Croatian Cereals

**DOI:** 10.3390/microorganisms9020216

**Published:** 2021-01-21

**Authors:** Marija Kovač, Mateja Bulaić, Jasna Jakovljević, Ante Nevistić, Tomislav Rot, Tihomir Kovač, Ivana Dodlek Šarkanj, Bojan Šarkanj

**Affiliations:** 1 Inspecto Ltd., Industrijska Zona Nemetin, Vukovarska Cesta 239b, 31000 Osijek, Croatia; marija.kovac@inspecto.hr (M.K.); mateja.bulaic@inspecto.hr (M.B.); jasna.jakovljevic@inspecto.hr (J.J.); ante.nevistic@inspecto.hr (A.N.); tomislav.rot@inspecto.hr (T.R.); 2 Faculty of Food Technology, Josip Juraj Strossmayer University of Osijek, Franje Kuhača 20, 31000 Osijek, Croatia; 3 Department of Agrobiotechnology (IFA-Tulln), Institute of Bioanalytics and Agro-Metabolomics, University of Natural Resources and Life Sciences Vienna (BOKU), Konrad Lorenzstr. 20, 3430 Tulln, Austria; 4 Department of Food Technology, University North, Trg dr. Žarka Dolinara 1, 48000 Koprivnica, Croatia; idsarkanj@unin.hr (I.D.Š.); bsarkanj@unin.hr (B.Š.)

**Keywords:** mycotoxins, pesticide residues, heavy metals, occurrence data, food and feed safety

## Abstract

Cereals are still one of the most important food and feed sources, thus determining cereal’s safety, i.e., compliance with legislation, is extremely important. As systematic investigations of nowadays unavoidable secondary fungal metabolites and other common legally regulated contaminants occurrence in Croatian cereals are still lacking, this research aims to monitor the contamination levels of nation-wide crops by mycotoxins, pesticide residues, and heavy metals by employing UHPLC-MS/MS, GC-MS/MS, and atomic absorption spectrometer (AAS) validated analytical methods. The most common secondary fungal metabolites were found to be *Fusarium* mycotoxins, with DON being the most occurring present in 73.7% of the samples. At least one pesticide residue was found in 331.8% of the samples, and Hg and Cd were the most occurring heavy metals. A total of 8.5% of the samples was non-compliant to the European Union (EU) legislation for food regarding the found mycotoxins concentrations, 4.5% regarding pesticide residues and none regarding heavy metals. The unusual presence of certain pesticide residue and heavy metal indicates the importance of systematic control of the contaminant presence, in order to gather enough occurrence data for proper risk assessment that these contaminants represent for the consumer’s health.

## 1. Introduction

The safety of cereals, and thus the quality, is of great importance since they represent one of the world’s most important source of food and feed [1]. In Republic of Croatia, according to the Paying Agency for Agriculture, Fisheries and Rural Development, maize and wheat are the most represented agricultural crops, followed by barley, oats, rye, and triticale, which are usually less cultivated. Cereal safety can be compromised by various contaminants, entering any point of food chain, including production, storage, processing, and transport. Treatment of crops with plant protective agents may leave pesticides residues in products of plant origin and indirectly animal origin. Natural toxins, such as mycotoxins, may be biosynthesized in feed- and foodstuff, while heavy metals can be introduced through water, air, or soil, thus contaminating cereals and products thereof [2]. When assessing the risks associated with cereal consumption, mycotoxins, pesticide residues and heavy metals need to be regularly monitored from the field to end-use product and their interaction must be taken into account [3,4]. If further genetic traits of individuals are taken into account with all xenobiotics that it was exposed to, the exposome can be characterized [5,6,7].

Mycotoxins are secondary metabolites of low-amount toxicity produced by various toxigenic fungi species in a field and/or during storage, most important of them being *Aspergillus*, *Fusarium*, *Alternaria*, and *Penicillium* [8,9]. The most relevant mycotoxins for agricultural production worldwide are aflatoxins B1 (AFT B1), B2 (AFT B2), G1 (AFT G1), and G2 (AFT G2), deoxynivalenol (DON), fumonisins B1 (FB1) and B2 (FB2), zearalenone (ZEN), T-2 and HT-2 toxins, and ochratoxin A (OTA), regulated within the European Union (EU) via Commission Regulation (EC) No 1881/2006 [10](EC, 2006a), Commission Recommendation 2013/165/EU [11] (EC, 2013), Commission Recommendation 2006/576/EC [12] (EC, 2006b) and Directive 2002/32/EC [13] (EC, 2002a) in the terms of the highest permitted levels in certain foodstuff and feedstuff. The mycotoxin species and concentrations are found to be in a correlation with meteorological conditions in the year of crop harvest, thus some species prefer warm weather and moisture, while the other prefer hot and drought season [14,15,16,17,18,19,20].

Plant protective agents containing different active substances are frequently used in agriculture to protect plants from insects, pests and pathogens, as well as to improve the amount and quality of harvest [21]. As a result, it is possible that pesticide residues accumulate in both raw materials and food products, possessing various toxic effects on human and animal health [22,23]. Within the EU, pesticide residues levels in certain food and feed of plant and animal origin have been set by the Commission (EC) No 396/2005. The information regarding their maximum residue limit (MRL) and toxicity is also included in the EU Pesticide database of the European Commission [24].

Heavy metals contamination of crops arises from irrigation with contaminated water, the usage of fertilizers and metal-based plant protective agents, rapid industrialization and urbanization of agricultural regions [21,25]. Heavy metals, including lead (Pb), cadmium (Cd), arsenic (As), and mercury (Hg), have no biological roles in a living organism and may induce numerous toxic effects, especially in children and embryos [25,26]. Accordingly, their concentrations in certain foodstuff and feedstuff are regulated within the EU via Commission Regulation (EC) No 1881/2006 [10] and Directive 2002/32/EC [13], setting the highest permitted levels in mg/kg.

Considering possible harmful effects of those contaminants groups, the usage of confirmatory analytical techniques such as liquid and gas chromatography (LC and GC) or inductively coupled plasma (ICP) coupled to (tandem) mass spectrometry (MS) in food safety control is crucial for unambiguous identification and quantification [27]. If such instruments are not available, the use of other instruments, as in our case the atomic absorption spectrometer (AAS) for the determination of metals, may be acceptable as long as they have satisfactory performance. However, mass spectrometry is preferred in a multi-contaminant analysis of food and feed. Due to its selectivity, mass spectrometry requires no extensive clean-up steps and is particularly useful for simultaneous determination of multiple compounds which ensures fast, easy and inexpensive determination, and at the same time, analytical parameters that meet the quality criteria required by regulations [28].

The toxicological relevance of the above mentioned contaminants and residues is well established, however the total health impact of combined exposure to various mycotoxins, pesticide residues and heavy metals, which is nowadays unavoidable, is difficult to assess, and thus remains largely unknown [3,4,29]. Monitoring the incidence of these pollutants is crucial for proper health risk assessment, especially if considering the possibility of their combined (possibly synergistic) effect, as showed for the combination of mycotoxins, pesticides (chlorpyriphos, pyrimiphos-methyl) and heavy metals (Cd, As) [3]. As systematic investigations of their occurrence in Croatian cereals are still lacking, this research aims to monitor the multiple contamination levels of nation-wide crops by mycotoxins, pesticide residues and heavy metals. In addition, it is set to compare the found levels of contaminants to the tolerance values set by the EU legislation and determine compliance/non-compliance of the tested cereal samples.

## 2. Materials and Methods

### 2.1. Sample Collection

Samples of cereals characteristic for the climate of the Republic of Croatia were selected to analyze and determine the occurrence of certain contaminants. A total of 118 cereal samples grown in the Croatian fields, including maize (61) and wheat (57), were collected from all Croatian counties, making sure that at least one sample originates from each county and the rest according to the representation, i.e., planting of agricultural areas with a certain cereal type. The sampling was carried out in warehouses/silos by trained manufacturer’s personnel in accordance with the methods set out in Commission Regulation (EC) No 401/2006 [30]. Upon arrival at the laboratory, the samples were briefly stored (up to 48 h) in a dry and dark place until milling to avoid subsequent production of mycotoxins as well as sample degradation in general. Samples were ground before analysis to obtain a homogeneous particle size (sieve pore size 0.5 mm) using Romer RAS mill (Romer Labs, Austria). After milling, the samples were stored in a freezer at −18 °C until analysis. All determination procedures applied for mycotoxins, pesticide residues and heavy metals analysis were previously successfully in-house validated and fitted to purpose.

### 2.2. Chemicals, Reagents, and Standards

Certified standards of mycotoxins were produced by Romer Labs Biopure (Romer Labs, Tulln, Austria), while certified pesticide and heavy metal standard solutions were obtained from CPAchem (CPAchem, Stara Zagora, Bulgaria). All standard solutions were stored according to manufacturer’s instructions and tempered to room temperature before use. LC-MS and HPLC grade acetonitrile and methanol were produced by J.T. Baker (J.T. Baker, Deventer, The Netherlands). LC-MS grade formic acid, LC-MS ammonium formate, nitric acid (65.0%) of trace analysis grade and hydrogen peroxide (30%) of extra purity were produced by Sigma–Aldrich (Sigma-Aldrich, St. Louis, MO, USA). Ultrapure water was generated by Niro VV system (Nirosta d.o.o., Osijek, Croatia). A QuEChERS (Quick, Easy, Cheap, Effective, Rugged, and Safe) buffer-salt mixture was produced by Agilent (Agilent Technologies, Santa Clara, CA, USA) and each portion consisted of 1 g trisodium citrate dihydrate, 1 g sodium chloride, 0.5 g disodium hydrogen citrate sesquihydrate, and 4 g of anhydrous magnesium sulfate. dSPE salt mixture was obtained from the same producer and consisted of 900 mg of anhydrous magnesium sulfate, 150 mg of primary secondary amine, and 150 mg graphitized carbon black.

### 2.3. Mycotoxins Determination

A dilute and shoot multimycotoxin LC-MS/MS method was used to determine 11 mycotoxins regulated in cereals by the above-mentioned EU legislative: AFB1, AFB2, AFG1, AFG2, DON, FB, FB2, ZEA, T-2, HT-2, and OTA. Sample preparation procedure used for analysis was adapted from Sulyok et al., (2020) [28]. Sample portion of 5 *g* was weighed into centrifuge tubes and extracted by solvent mixture 79/20/1 acetonitrile/ultrapure water/formic acid (*v/v/v*) using mechanical shaker for 90 min. Aliquot of the raw extract was afterwards diluted using 20/79/1 acetonitrile/ultrapure water/formic acid (*v/v/v*), filtered through 0.22 µm nylon filter and without further clean-up injected into a LC-MS/MS system. Waters Acquity H-class UPLC system (Waters, Milford, MA, USA) was employed to perform chromatographic separation of selected mycotoxins by using a HSS T3 column (100 × 2.1 mm, 1.8 µm particle size) (Waters, Milford, MA, USA) maintained at 40 °C. Gradient elution was carried out with eluent A consisting of an aqueous solution of ammonium formate (5 mM) and eluent B being methanol at constant flow rate of 0.3 mL/min. The volume of injected sample was 10 µL. The separation started with 95% A followed by linearly decrease to 50% A in 6 min and in the next 4 min to 5% A with a hold time of 5 min, afterwards switching to 95% A and column equilibration to initial conditions in the next 3 min. The UPLC system was coupled to Waters Xevo TQD tandem mass spectrometer (Waters, Milford, MA, USA) equipped with an electrospray ionization interface (ESI) operating in both positive and negative ionization mode. ESI-MS/MS analysis was performed in multiple reaction monitoring (MRM) mode meaning that for each compound at least one precursor and two product ions were used for both identification and quantification purposes, selecting the most abundant product ion for quantification and the second one for confirmation purposes. Cone voltage and collision energy values were optimized for each precursor ion and different product ions. The ionization source parameters were: capillary voltage 1.5 kV, extractor voltage 3 V, source temperature 150 °C, desolvation temperature 400 °C, cone gas flow 0 L/h, and desolvation gas flow 650 L/h (both gases were nitrogen). Collision-induced dissociation was performed using argon as collision gas at a pressure of 4 × 10^−3^ mbar in the collision cell. Data acquisition was performed using MassLynx and TargetLynx software (v. 4.1., Waters, Milford, MA, USA). The MRM transitions for each mycotoxin and the applied cone voltages and collision energies are summarized in Table A1 in Appendix A.

Upon mycotoxin content determination and data analysis, the most relevant cereal samples containing very high or very low mycotoxin levels were selected (at least one from each county) and further subjected to pesticides and heavy metals analysis.

### 2.4. Pesticide Residues Determination

Standardized QuEChERS method (EN 15662) was employed in sample preparation procedure for pesticide residues (listed in Table A2 and Table A3, Appendix A) determination. Samples (portions of 5 g) were weighed into 50 mL centrifuge tubes and soaked in 10 mL of ultrapure water, followed by 1 min extraction using 10 mL of acetonitrile and 1 min extraction by addition of QuEChERS buffer-salt mixture. Sample tubes were then centrifuged for 5 min at 3000× *g* at room temperature using Restek Q-sep 3000 centrifuge (Restek, Bellefonte, PA, USA). An aliquot of the organic phase (6 mL) from each sample was transferred into a 15 mL centrifuge tube containing dSPE salt mixture, shaken vigorously for 0.5 min and afterwards centrifuged at 3000× *g* for 5 min at room temperature. The clean extract was filtered through 0.22 µm nylon filter, transferred to a glass vial, and finally analyzed by LC-MS/MS and GC-MS/MS.

For LC-MS/MS determination, Xevo TQD tandem mass spectrometer (Waters, USA) equipped with ESI source and Acquity H-class UPLC system (Waters, USA) was used. For chromatographic separation Ultra Aqueous C18 column (100 × 2.1 mm, 3 µm particle size) (Restek, USA) with a gradient elution consisting of eluent A (aqueous 4 mM ammonium formate/0.1% formic acid) and eluent B (4 mM ammonium formate/0.1% formic acid in methanol) was used. The analysis started with 90% A, held for 1.5 min, increased to 60% B in 2.5 min and in the next 4 min to 70% B, and then to 100% B in 3 min with a 1 min hold, followed by 3 min return to initial conditions, to give a total run time of 15 min. The flow rate was set to 0.5 mL/min and the column temperature was maintained at 50 °C, while the injection volume was 10 µL. For analyte detection, MRM mode was applied and for each analyte at least two transitions were monitored (Table A2). The capillary voltage was 3.5 kV, the source temperature was 150 °C, and the desolvation gas temperature was 400 °C. The desolvation gas flow was 650 L/h, while the cone gas flow was 10 L/h (both nitrogen). Collision-induced dissociation was performed using argon as the collision gas at a pressure of 3.7 × 10^−3^ mbar in the collision cell. The MassLynx and TargetLynx software (v. 4.1., Waters, USA) were used for the instrument control, data acquiring, and processing.

GC-MS/MS used for pesticide determination was Trace 1300 gas chromatograph coupled to TSQ 8000 Evo tandem mass spectrometer (Thermo Scientific, Waltham, MA, USA) equipped with split/splitless injector operating in a splitless mode (40 mL/min) at 200 °C with injection volume of 1 µL and TG-XLBMS chromatographic column (30 m × 0.25 mm × 0.25 µm) (Thermo Scientific, USA). The instrument control, data acquisition, and processing were conducted by TraceFinder software (v. 3.3, Thermo Scientific, Waltham, MA, USA). The gas carrier was helium (6.0) maintained at constant flow of 1.2 mL/min. Temperature program used for chromatographic separation was ranging from 40 to 300 °C in four stages as follows: starts with 40 °C (hold time 1.5 min), rises with rate of 25 °C/min to 90 ° C (1.5 min hold) and then to 180 °C (0 min hold), 5 °C/min to 280 °C (0 min hold) and finally with 10 °C/min to 300 °C (hold 10 min). For ionization, electron impact ionization in positive mode was applied, with ion source temperature of 300 °C and MS transfer line temperature of 250 °C. MRM mode was used for analyte detection, with the MS conditions optimized for each compound, as stated in Table A3.

### 2.5. Heavy Metals Determination

A Perkin Elmer AAnalyst 600 was used for determination of traces of heavy metals including Pb, Cd, As, and Hg. For the analysis of Pb, Cd, and As, the graphite furnace performance was used, while for Hg analysis hydride technique was applied. Sample preparation procedure was based on sample degradation using microwave-assisted digestion and nitric acid (HNO_3_)/hydrogen peroxide (H_2_O_2_) as digestion reagents. Samples were accurately weighed by using plastic equipment to avoid cross-contamination (portion of 0.5 g) into microwave tubes, 7 mL of 65% HNO_3_, and 2 mL of H_2_O_2_ were added to each sample, followed by microwave digestion. Digestion program included 15 min heating procedure to 210 °C, holding at the achieved temperature for 20 min and cooling for the next 10 min. Afterwards, samples were quantitatively transferred to volumetric flask by rinsing the tubes with ultrapure water. Aliquots (1 µL) of prepared cereal samples were injected into the AAS. The instrument operating conditions for the determination of heavy metals were as follows: Pb-wavelength 283.3 nm, slit width 0.7 nm; Cd-wavelength 228.8 nm, slit width 0.7 nm; As-wavelength 193.7 nm, slit width 0.7 nm; Hg-wavelength 253.7 nm, slit width 0.7 nm.

### 2.6. Method Validation

Method validation for was carried according to the requirements of the accreditation standard ISO/IEC 17025 standard and relevant EU legislation [27,30,31,32].

Accuracy and precision for each method were estimated by means of recovery experiments at different spiking levels. Method procedures were applied to blank cereal samples spiked at three different concentration levels of each analyte and each sample was prepared in triplicate and injected in triplicate. Method accuracy was expressed as average recovery (R%) and method precision (measurement repeatability) as relative standard deviation (RSD%). The obtained results were satisfactory with most of the recoveries being between 70% and 120% and RSD below 20%. Validation data on representative cereal (wheat), including achieved limits of detection (LOD) and quantification (LOQ) is shown in Table A4, Table A5 and Table A6. Beside in-house validation, methods were also confirmed externally (measurement reproducibility) by successful participation in a proficiency testing schemes expressed by z-scores within range |z| ≤ ± 2 presented in Table A7 in Appendix B.

## 3. Results and Discussion

A total of 118 samples of Croatian cereals were collected from all twenty Croatian counties and analyzed for mycotoxins contamination levels, with the most significant samples regarding the mycotoxin content submitted to pesticides residues and heavy metals analyses. Previously in-house validated methods, fitted for purpose, were employed for all the contaminants determination procedures. The found compounds were quantified using external calibration (linear calibration polynomial type) in a solvent or appropriate matrix, with the measured concentrations corrected for method recovery analyzed within each batch if not within the range of 90–110% allowed for mycotoxins by Commission Regulation (EC) No 401/2006 [30], and within the range 70–120% allowed for pesticide residues by SANTE/11813/2017 [31].

### 3.1. Mycotoxins in Croatian Cereals

**Figure 1:**
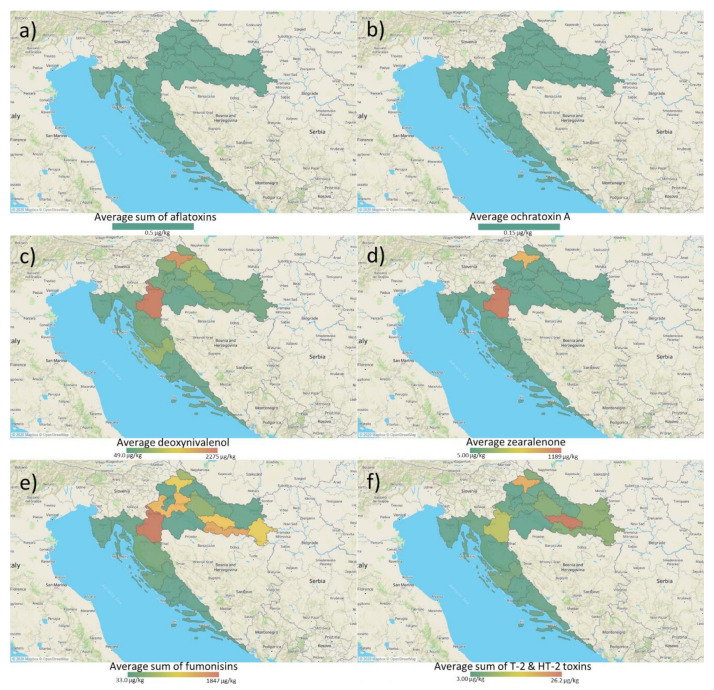
Distribution of regulated mycotoxin occurrence in Croatian cereals: (**a**) sum of aflatoxins (not detected); (**b**) ochratoxin A; (**c**) deoxynivalenol; (**d**) zearalenone; (**e**) sum of fumonisin B1 and B2; (**f**) sum of T-2 and HT-2 toxins. The map shows the averages of detected mycotoxins per county.

The results of mycotoxin occurrence investigation are shown in the Table 1. The most common mycotoxins found in all collected samples of different cultures at concentrations above LOD were *Fusarium* mycotoxins. The incidence of mycotoxins found in all analyzed samples was as follows: DON 73.7% (63–4902 µg/kg), HT-2 45.8% (3–41 µg/kg), FB1 43.2% (61–9344 µg/kg), ZEA 36.4% (10–2068 µg/kg), FB2 33.9% (49–2442 µg/kg), T-2 16.9% (3–73 µg/kg), while AFB1, AFB2, AFG1, AFG2, and OTA were not detected. The average value of all positive samples for the presence of the most common mycotoxin DON was 589 µg/kg, and the highest value of 4902 µg/kg was found in the maize sample. A total of 10 cereal samples (all maize) did not comply with the highest permitted values for mycotoxins in foodstuff given in Commission Regulation (EC) No. 1881/2006 [10] and Commission Recommendation 2013/165/EU [11]. Distribution of mycotoxin occurrence is shown in Figure 1.

The dominant occurrence of DON in cereals from Croatian field was confirmed by other authors. Španić et al. (2019) [18] investigated *Fusarium* head blight in different wheat varieties of the seasons 2015/2016 and 2016/2017. Among determined *Fusarium* mycotoxins, DON was quantified in all of the control samples. Habshied et al. (2019) [19] studied the occurrence of regulated and unregulated *Fusarium* mycotoxins in six barley cultivars during the years 2016–2018. Among investigated mycotoxins, DON proved to be the most dominant, however, found levels were not exceeding the EU regulation. Pleadin et al. (2017) [33] investigated the occurrence of selected regulated mycotoxins in Croatian cereals of 2015 harvest. DON was found to be the most frequent mycotoxin and maize the most contaminated cereal with 46% of samples non-compliant to the EU legislation. According to the authors, occurrence and found levels of mycotoxins can be correlated with weather conditions varying from year to year: in the case of *Fusarium* mycotoxins, their occurrence is favored by periods of rain accompanied with moderate air temperature during plant flowering. Earlier studies showed significant contamination of Croatian cereals and feedstuff with aflatoxins, namely AFB1, associated with climatic conditions, i.e., periods of extremely hot and dry weather during the critical stages of plant growth [16,34,35].

### 3.2. Pesticide Residues in Croatian Cereals

**Figure 2:**
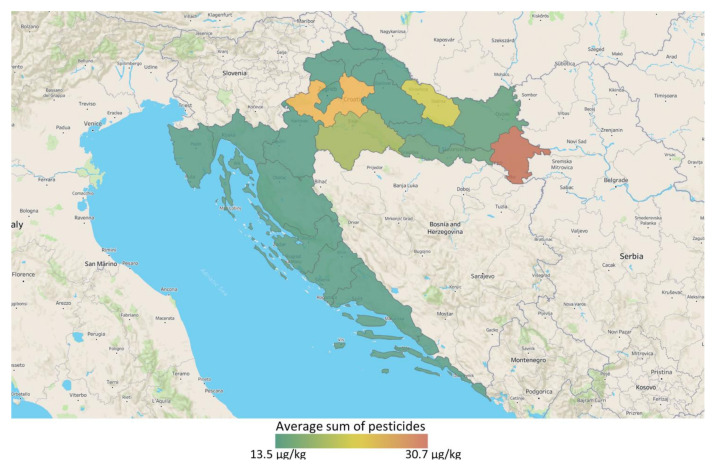
Occurrence of pesticides in Croatian cereals. The map shows the averages of sum of all detected pesticides per County.

Out of 333 analyzed substances, at least one pesticide residue was found and quantified in 31.8% of the cereal samples tested for pesticides, including fungicides carboxin, epoxiconazole and tebuconazole, insecticides chlorpyrifos, cypermethrin, pirimiphos-methyl, thiamethoxam, and thiofanox, as well as pesticide synergist piperonyl butoxide (Table 2). Piperonyl butoxide and cypermethrin were the most occurring substances both present in 9.1% of the analyzed samples in concentrations of 0.015–0.028 mg/kg and 0.055–0.058 mg/kg, respectively, followed by epoxiconazole (0.020), pyrimiphos-methyl (0.021), carboxin (0.077 mg/kg), tebuconazole (0.010 mg/kg), chlorpyrifos (0.010 mg/kg), thiamethoxam (0.010 mg/kg), and thiofanox (0.083 mg/kg) present in 4.5% of all cereal samples. Distribution of sum of pesticide residues occurrence is represented in Figure 2. The sum of pesticide residue was used, as there was always maximum one pesticide positive sample per county.

The sample with highest pesticide residues contamination was maize, containing four of the nine quantified substances: thiofanox at 0.083 mg/kg, tebuconazole 0.010 mg/kg, thiamethoxam 0.010 mg/kg, and carboxin 0.077 mg/kg. Out of all analyzed samples, the mentioned maize sample, originating from Vukovar-Srymia county, did not comply with the EU legislation regarding the maximum pesticide residue level permitted [24], containing elevated level of insecticide thiofanox (0.083 mg/kg).

The (elevated) presence of residue thiofanox is considered unusual, since it is an unapproved active substance according to the EU Pesticide database and plant protection agent containing thiofanox is not on the List of registered plant protection products according to the Croatian Ministry of Agriculture. Piperonyl butoxide, a semi-synthetic synergist, is particularly used in natural pyrethrum and synthetic pyrethroid insecticides, but it can occur in other agents and as such it is used for crop treatments, stored grain protection, disinfestation of grain storage facilities and indoor uses [36], thus having a possibility of contaminating cereal-based food. Its presence in various products clearly indicates the usage of plant protective agents, which is particularly important in the control of organic food production. In cereal samples, piperonyl butoxide was co-occurring with insecticide pyrimiphos-methyl. The quantified fungicides carboxin, epoxiconazole, and tebuconazole are found to affect the growth of certain mycotoxigenic fungi, and thus the occurrence of certain mycotoxins [37]. The fact that the analyzed samples containing the above-mentioned active substances were contaminated with low concentrations of the regulated mycotoxins confirms the finding of their fungicidal action. The occurrence of pyrimiphos-methyl and chlorpyriphos in wheat is especially worrying since they have shown high cytotoxinc effect in study by Clarke et al. (2015) [3].

Investigation of the pesticide residues in foodstuff from Croatian markets, including cereals and products thereof, conducted by Miloš et al. (2014) [38], showed a small percentage of non-compliant samples regarding the EU established MRLs, and none of the cereals samples. Pesticide residue levels in foods on the European market is annually reported by EFSA, based on data from the official national control activities carried out by EU Member States, Iceland, and Norway, and includes a subset of data from the EU-coordinated control program. In 2016, out of 608 cereal sample analyzed, 34.9% contained one or several pesticides in quantifiable concentrations. The residue concentrations exceeded the MRLs in 0.7% of the samples, containing pirimiphos-methyl, permethrin, hexaconazole or dichlorvos. In total, 19 different pesticide residues were quantified [24]. For year 2017, EFSA (2019) [39] reported multiple residues in 12.9% of analyzed rye samples, and 16.3% of analyzed rice samples. The residue concentrations exceeded the MRLs in 5.1% of the rice samples for 12 different pesticides, including not approved active substances acephate, carbendazim, hexaconazole, methamidophos, permethrin, profenofos, and triazophos. In rye samples, the residue concentrations exceeded the MRLs in 1.9% of the analyzed samples, containing chlorpyrifos, glyphosate, permethrin, pirimiphos-methy, and tebuconazole. A total of 676 unprocessed cereal samples were analyzed in 2018, of which 24.7% were characterized as non-compliant, with active substance tricyclazole being the most occurring [40]. Comparing the abovementioned data with the occurrence data gathered in our investigation, insecticides were the most commonly found pesticide residues in cereals in both cases, and often the reason for exceeding the EU MRLs. Even though a significant occurrence of pesticide residues was observed (up to 34.9%), only a small percentage of samples was reported as non-compliant, roughly to the value of 5%, which is in accordance with our investigation results.

### 3.3. Heavy Metals in Croatian Cereals

**Figure 3:**
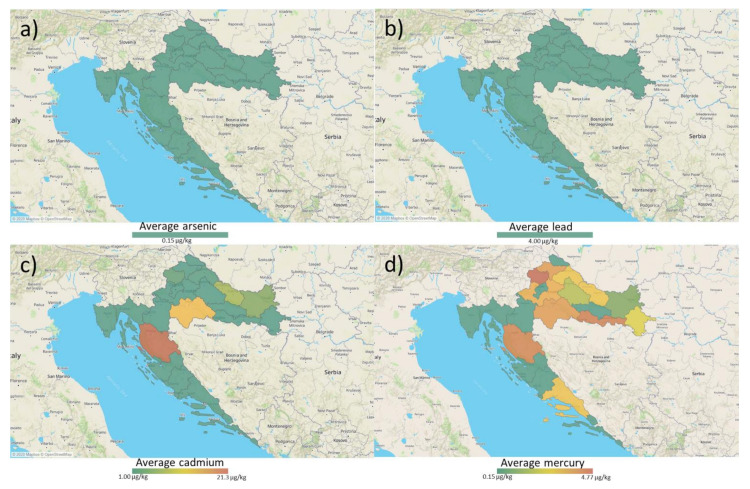
Distribution of heavy metals occurrence in Croatian cereals: (**a**) Arsenic (below limit of detection); (**b**) Lead (below limit of detection; (**c**) Cadmium; (**d**) Mercury. The map shows the averages of detected heavy metals per County.

The majority (90.9%) of cereal samples analyzed for heavy metals contained Hg in concentrations above LOQ (0.009–0.016 mg/kg), with highest concentrations found in analyzed maize samples. Cd was quantified in 27.3% of samples (0.007–0.080 mg/kg), all of which were wheat originating from all Croatian regions, while Pb and As were not found. Regarding the determined concentrations of heavy metals, all of the analyzed cereal samples complied with the highest permitted levels for cereals set by the EU legislation [10], which are 0.20 mg/kg for Pb, 0.10, and 0.20 mg/kg for Cd. Although levels of Hg and As are not regulated for cereals, they are often sought to be analyzed when food and feed safety is to be determined. The results of heavy metals occurrence investigation are represented in Table 3 and the County specific occurrence data distribution shown in Figure 3.

According to the annual reports of Rapid Alert System for Feed and Feed for years 2016, 2017 and 2018, Hg, Pb and Cd are predominately notified [41,42,43], however the risk posed by heavy metals is mainly related to fish and fish products. To the best of our knowledge, data on heavy metals occurrence in Croatian cereals are lacking.

## 4. Conclusions

An occurrence investigation study of mycotoxins, pesticide residues, and heavy metals in Croatian cereals was performed using validated analytical UHPLC-MS/MS, GC-MS/MS and AAS methods. *Fusarium* mycotoxins were the most common mycotoxins found in all collected samples of different cultures, with DON being the most frequent mycotoxin occurring in the 73.7% of samples, while aflatoxins and OTA were not found. Among heavy metals, Hg and Cd were the most occurring, while Pb and As were not detected. A total of 31.8% of samples analyzed for pesticides contained at least one pesticide residue, with synergist piperonyl butoxide and insecticide cypermethrin being the most occurring residues each present in 9.1% of the samples.

Only a small percentage of the samples was non-compliant to the EU legislation for food: 8.5% regarding mycotoxins, 4.5% regarding pesticide residues, but none regarding heavy metals. In addition, the unusual presence of thiofanox, not approved active substance according to the EU Pesticide database, and the unusual presence of Hg in all analyzed samples was recorded. Accordingly, the results of the conducted multi-contaminant analysis of Croatian cereals indicate the importance and necessity of constant and continuous control of food and feed safety, including contaminant analysis, in order to collect enough occurrence data for proper risk assessment that these contaminants represent for the consumer’s health.

This very valuable data can serve as the identified mixture of food contaminants for further toxicological studies and better risk assessment for the consumers.

## Figures and Tables

**Table 1:** Occurrence of regulated mycotoxins in Croatian cereals.

Compound	Maize (*n* = 61)		Wheat (*n* = 57)	
	F% (*n*)	Median conc. µg/kg (min.–max. conc.)	F% (*n*)	Median conc. µg/kg (min.–max. conc.)
Aflatoxin B1	<LOD	<LOD	<LOD	<LOD
Aflatoxin B2	<LOD	<LOD	<LOD	<LOD
Aflatoxin G1	<LOD	<LOD	<LOD	<LOD
Aflatoxin G2	<LOD	<LOD	<LOD	<LOD
Deoxynivalenol	78.7 (48)	356 (68–4902)	68.4 (39)	175 (63–867)
Fumonisin B1	83.6 (51)	427 (61–9344)	<LOD	<LOD
Fumonisin B2	65.6 (40)	170 (49–2442)	<LOD	<LOD
Zearalenone	73.8 (43)	21 (10–2068)	<LOD	<LOD
T-2 toxin	32.8 (20)	4 (3–73)	<LOD	<LOD
HT-2 toxin	88.5 (54)	5 (3–41)	<LOD	<LOD
Ochratoxin A	<LOD	<LOD	<LOD	<LOD

*n*—number of samples; F—frequency (occurrence), number of samples with mycotoxin concentration above detection limit; <LOD—below detection limit.

**Table 2:** Occurrence of the found pesticide residues in Croatian cereals.

Compound	Maize (*n* = 15)		Wheat (*n* = 7)	
	F% (*n*)	Median conc. mg/kg (min.–max. conc.)	F% (*n*)	Median conc. mg/kg (min.–max. conc.)
Piperonyl butoxide	13.3 (2)	0.022 (0.015–0.028)	<LOD	<LOD
Chlorpyriphos	<LOD	<LOD	14.3 (1)	0.010
Epoxiconazole	<LOD	<LOD	14.3 (1)	0.020
Thiofanox	6.7 (1)	0.083	<LOD	<LOD
Tebuconazole	6.7 (1)	0.010	<LOD	<LOD
Thiametoxam	6.7 (1)	0.010	<LOD	<LOD
Carboxin	6.7 (1)	0.077	<LOD	<LOD
Cypermethrin	6.7 (1)	0.055	14.3 (1)	0.058
Pyrimiphos-methyl	6.7 (1)	0.021	<LOD	<LOD

*n*—number of samples; F—frequency (occurrence), number of samples with mycotoxin concentration above detection limit; <LOD—below detection limit.

**Table 3:** Heavy metals occurrence in Croatian cereals.

Compound	Maize (*n* = 15)		Wheat (*n* = 7)	
	F% (*n*)	Median conc. mg/kg (min.–max. conc.)	F% (*n*)	Median conc. mg/kg (min.–max. conc.)
Lead	<LOD	<LOD	<LOD	<LOD
Cadmium	<LOD	<LOD	85.7 (6)	0.042 (0.007–0.080)
Arsenic	<LOD	<LOD	<LOD	<LOD
Mercury	86.7 (13)	0.014 (0.011–0.016)	100.0 (7)	0.012 (0.009–0.014)

*n*—number of samples; F—frequency (occurrence), number of samples with mycotoxin concentration above detection limit; <LOD—below detection limit.

## Data Availability

The data presented in this study are openly available.

## Appendix A

**Table A1:** UHPLC–MS/MS parameters optimized for mycotoxin determination.

Compound Name	Retention Time (min)	Precursor Ion (*m*/*z*)	Cone Voltage (V)	Primary Production (*m*/*z*)	Collision Energy (V)	Secondary Production (*m*/*z*)	Collision Energy (V)
Aflatoxin B1	8.7	313.0	60	241.0	38	285.0	23
Aflatoxin B2	8.5	315.0	60	259.0	30	287.0	25
Aflatoxin G1	8.1	329.0	60	243.0	28	311.0	24
Aflatoxin G2	7.8	331.0	60	313.0	24	313.0	24
Deoxynivalenol	4.7	297.0	25	231.0	25	249.0	10
Fumonisin B1	9.4	722.4	20	334.3	40	352.3	40
Fumonisin B2	10.4	706.4	50	336.2	40	318.2	40
Zearalenone	10.5	317.1	−58	131.0	−30	175.0	−20
T-2 toxin	10.1	484.7	25	185.0	30	215.0	20
HT-2 toxin	9.6	442.6	25	263.4	10	215.3	15
Ochratoxin A	9.5	404.1	30	215.3	15	239.0	24

**Table A2:** UHPLC–MS/MS parameters optimized for pesticide residues determination.

Compound Name	Retention Time (min)	Precursor Ion (*m*/*z*)	Cone Voltage (V)	Primary Production (*m*/*z*)	Collision Energy (V)	Secondary Production (*m*/*z*)	Collision Energy (V)
Acephate	1.7	184.1	18	49.0	18	143.0	8
Acetamiprid	4.8	223.0	23	126.0	20	56.1	15
Acibenzolar-S-metil	6.6	210.9	43	135.9	29	91.0	20
Aldicarb	4.9	208.0	16	116.1	6	89.1	12
Aldicarb sulfone	3.3	240.0	18	148.0	14	86.0	20
Aldicarb sulfoxide	3.5	207.0	24	132.0	6	89.0	12
Ametryn	6.0	228.1	45	186.1	18	96.0	30
Aminocarb	3.0	209.1	35	152.0	14	137.1	25
Amitraz	7.8	294.0	40	148.0	18	91.1	45
Azoxystrobin	6.4	404.1	25	372.0	15	344.0	25
Benalaxyl	7.8	326.1	30	148.0	20	91.0	35
Bendiocarb	5.2	224.1	24	167.0	8	109.0	18
Benzoximate	8.3	364.0	18	199.1	10	105.0	20
Bifenazate	6.8	301.1	25	198.0	8	170.0	20
Bitertanol	8.4	338.1	15	99.1	16	269.2	10
Boscalid	6.6	342.9	35	307.0	18	139.9	18
Bromuconazole 1	7.0	378.0	40	159.0	25	70.0	20
Bromuconazole 2	8.1	378.0	40	159.0	25	70.0	20
Bupirimate	7.1	317.0	32	108.0	30	166.0	26
Buprofezin	9.3	306.1	26	201.0	11	116.2	16
Butafenacil	6.8	492.0	25	331.0	25	180.0	45
Butocarboxim	4.9	213.0	35	75.0	15	116.0	10
Butoxycarboxim	3.2	223.0	27	106.0	8	166.0	8
Carbaryl	5.5	202.0	24	145.0	10	127.0	24
Carbendazim	3.9	192.1	35	160.1	17	132.1	28
Carbetamide	5.0	237.0	22	192.0	8	118.0	12
Carbofuran	5.3	222.1	26	165.1	12	123.0	21
Carbofuran-3-hidroxy	4.4	238.0	27	163.0	16	181.0	11
Carboxin	5.4	236.0	32	143.0	16	87.0	24
Carfentrazone-ethyl	7.4	412.0	45	346.0	20	366.0	15
Chlorantraniliprole	6.0	483.8	25	285.8	18	452.8	16
Chlorfluazuron	11.0	539.8	38	382.9	22	158.0	22
Chlorotoluron	5.8	213.0	33	72.0	16	46.0	15
Chloroxuron	7.2	291.1	36	72.0	18	218.1	25
Chlorpyrifos	9.9	349.9	29	97.0	32	198.0	18
Clethodim	8.8	360.0	26	164.0	20	268.1	11
Clofentezine	8.7	303.0	25	138.0	15	102.0	35
Clomazone	6.2	240.0	30	125.0	18	89.0	46
Clothianidin	4.4	250.0	25	169.0	15	132.0	17
Cyazofamid	7.2	325.0	24	107.9	13	261.0	10
Cycluron	6.2	199.0	36	89.1	14	69.2	21
Cymoxanil	4.6	199.0	20	128.0	8	111.0	18
Cyproconazole	6.8	292.2	32	70.2	20	125.1	25
Cyprodinil	7.5	226.0	40	93.0	33	77.0	38
Cyromazine	1.4	167.0	40	85.0	18	60.0	18
Desmedipham	5.8	318.1	25	182.0	10	154.0	22
Diclobutrazol	7.5	328.0	35	70.0	25	59.1	25
Dicrotophos	4.4	238.0	24	112.0	9	193.0	11
Diethofencarb	6.2	268.0	21	226.0	10	124.0	32
Difenoconazole	9.3	406.0	35	251.1	25	253.1	25
Diflubenzuron	7.4	311.1	30	158.1	14	141.0	30
Dimethoate	4.4	230.1	25	199.0	10	125.0	20
Dimethomorph	6.9	388.1	38	300.9	22	165.0	28
Dimoxystrobin	7.5	327.1	22	205.2	10	116.1	18
Diniconazole	8.4	326.0	45	70.2	25	159.0	35
Dinotefuran	3.3	203.0	22	129.0	14	157.0	6
Dioxacarb	4.5	224.1	25	123.1	17	167.1	8
Diuron	5.6	233.0	32	72.1	16	46.3	14
Doramectin	11.4	916.6	20	593.4	10	331.2	20
Emamectin benzoate b1a	10.4	886.6	70	158.0	40	126.0	40
Emamectin benzoate b1b	10.0	872.6	68	158.2	38	126.2	48
Epoxiconazole	7.5	330.0	30	101.0	45	121.0	25
Eprinomectin	11.1	915.6	20	186.0	35	154.0	35
Etaconazole	7.5	328.1	45	159.0	30	205.0	15
Ethiofencarb	5.5	226.1	20	107.0	15	164.0	8
Ethiprole	6.3	414.1	12	350.9	25	396.9	9
Ethirimol	4.9	210.1	48	140.0	22	98.0	28
Ethofumesate	6.1	287.1	36	121.1	16	259.1	9
Etoxazole	10.6	360.1	36	141.0	36	57.2	32
Famoxadone	8.0	392.2	20	331.1	8	238.0	18
Fenamidone	6.3	312.1	26	92.0	23	236.1	12
Fenarimol	7.1	331.0	45	268.0	22	81.0	32
Fenazaquin	11.3	307.2	35	57.2	22	161.0	15
Fenbuconazole	7.5	337.0	40	70.1	20	125.0	30
Fenhexamid	6.8	302.1	60	97.2	25	55.3	38
Fenobucarb	6.1	208.0	26	94.9	14	152.0	8
Fenoxycarb	7.4	302.1	30	116.1	10	88.0	17
Fenpropimorph	6.2	304.2	50	147.1	28	57.2	30
Fenpyroximat	11.0	422.2	35	366.1	15	138.1	35
Fenuron	4.5	165.0	27	71.9	16	45.9	14
Fipronil	7.0	436.8	42	367.8	16	290.0	26
Flonicamid	3.4	230.1	40	203.1	18	174.0	16
Fluazinam	9.7	462.7	−45	415.7	−20	397.8	−15
Flubendiamide	7.4	683.1	22	408.0	12	274.1	29
Fludioxonil	6.4	247.0	−42	126.0	−35	180.0	−28
Flufenacet	6.8	364.0	20	152.1	19	194.1	10
Flufenoxuron	10.7	489.1	36	158.0	22	141.0	48
Fluomethuron	5.7	233.2	34	72.2	16	46.4	15
Fluoxastrobin	7.2	459.0	40	427.0	15	188.0	38
Fluquinconazole	7.0	376.0	45	348.8	16	306.9	28
Flurochloridone	6.7	312.0	40	292.0	20	145.0	50
Flusilazole	7.6	316.0	40	247.0	20	165.0	25
Flutolanil	6.4	324.1	38	242.1	24	65.0	42
Flutriafol	5.7	302.1	30	70.2	18	123.1	35
Forchlorfenuron	5.9	248.1	32	129.0	16	93.0	31
Fuberidazol	4.6	185.0	45	157.0	22	65.0	40
Furalaxyl	6.2	302.1	25	95.0	25	242.0	15
Furathiocarb	9.5	383.2	30	194.9	18	252.0	12
Halofenozide	6.1	331.1	15	104.9	20	275.0	5
Hexaconazole	8.2	314.0	35	70.1	20	159.0	35
Hexythiazox	10.1	353.0	32	228.1	14	168.1	24
Hydramethylnon	10.6	495.2	78	323.3	30	151.1	60
Imazalil	5.7	297.0	40	159.0	18	69.0	20
Imidacloprid	4.6	256.1	26	209.1	20	175.1	18
Indoxacarb	8.8	528.0	32	150.0	25	218.0	24
Ipconazole	9.1	334.2	40	70.0	28	125.0	40
Iprovalicarb	6.6	321.1	26	119.1	18	203.1	8
Isocarbofos	5.7	307.1	12	230.9	15	291.1 > 231.1	13
Isoprocarb	5.6	194.1	24	95.1	14	137.1	8
Isoproturon	6.0	207.2	36	72.1	19	46.1	16
Ivermectin	11.6	892.6	15	307.2	20	569.4	15
Kresoxim-methyl	7.4	314.1	20	206.0	20	116.0	5
Linuron	6.5	249.1	35	160.0	18	182.0	16
Lufenuron	10.6	511.2	40	158.0	22	141.0	48
Mandipropamid	6.5	412.3	35	328.2	14	356.2	10
Mefenacet	7.0	299.0	28	148.0	14	120.0	26
Mepanipyrim	7.2	224.1	50	106.0	25	77.0	35
Mepronil	6.4	270.1	30	119.0	25	91.0	38
Mesotrione	4.8	340.1	38	104.0	30	228.1	18
Metaflumizone	10.1	507.1	54	178.1	22	287.1	22
Metalaxyl	5.8	280.1	26	220.1	14	192.1	18
Metconazole	8.3	320.1	36	70.0	22	125.0	35
Methabenzthiazuron	6.1	222.0	30	165.0	15	150.0	32
Methamidophos	1.3	142.0	28	93.9	14	124.9	14
Methiocarb	6.4	226.0	26	169.0	8	121.0	18
Methomyl	3.8	163.1	16	106.0	10	88.1	10
Methoprotryne	6.1	272.2	36	198.2	22	240.2	19
Methoxyfenozide	6.5	369.1	15	149.1	20	313.2	5
Metobromuron	5.8	259.1	31	148.1	15	170.0	19
Metolachlor	7.1	284.1	22	252.1	16	176.1	26
Metribuzin	5.2	215.0	38	187.1	19	84.1	19
Mevinphos	4.5	225.1	24	127.1	12	193.1	6
Mexacarbate	4.2	223.2	34	166.1	15	151.0	24
Monocrotophos	4.1	224.1	24	127.1	18	98.1	12
Monolinuron	5.6	215.0	30	126.0	17	148.0	14
Moxidectin	11.5	640.5	25	528.4	10	498.3	15
Myclobutanil	6.9	289.1	40	70.2	18	125.1	35
Neburon	7.5	275.0	35	88.0	16	114.0	14
Nitenpyram	3.7	271.1	35	224.9	12	125.9	35
Novaluron	9.8	493.0	34	158.0	22	141.0	44
Nuarimol	6.3	315.0	45	252.0	20	81.1	20
Omethoate	2.6	214.1	24	183.1	12	125.1	22
Oxadixyl	5.1	279.0	38	219.0	10	132.0	28
Oxamyl	3.7	237.0	15	72.0	12	90.0	8
Paclobutrazol	6.4	294.1	28	70.2	22	125.1	28
Penconazole	8.2	284.0	35	70.1	25	159.0	25
Pencycuron	8.4	329.1	35	125.0	22	218.0	15
Pendimethalin	10.0	282.2	18	212.2	12	194.1	18
Phenmedipham	6.0	301.2	32	168.0	8	107.9	32
Picoxystrobin	7.4	368.0	18	145.1	22	205.1	8
Piperonyl butoxide	9.8	356.3	20	176.9	10	119.0	36
Pirimicarb	5.3	239.1	34	72.0	21	182.1	17
Prochloraz	8.6	376.0	25	308.0	10	70.0	25
Promecarb	6.5	208.1	25	109.0	16	151.0	8
Prometon	5.7	226.0	42	142.0	22	86.3	28
Prometryn	6.5	242.0	45	158.0	22	200.1	18
Propamocarb	2.8	189.1	34	102.0	18	144.0	12
Propargite	10.3	368.2	22	231.1	10	175.1	16
Propham	5.5	180.0	14	138.0	8	120.0	16
Propiconazole	8.3	342.0	45	69.0	20	159.0	35
Propoxur	5.2	210.0	18	111.0	15	168.0	7
Prothioconazole	7.8	344.0	24	125.1	30	188.9	22
Pymetrozine	3.7	218.0	30	105.0	30	79.0	15
Pyracarbolid	5.3	218.1	32	125.1	20	97.1	28
Pyraclostrobin	8.3	388.1	24	193.9	12	163.0	26
Pyridaben	11.0	365.1	25	147.1	26	309.1	12
Pyrimethanil	6.4	200.0	52	107.0	24	82.0	28
Pyriproxifen	9.9	322.1	30	96.0	20	185.0	22
Quinoxyfen	10.4	308.0	65	197.0	30	161.9	45
Rotenone	7.6	395.0	45	213.1	25	192.1	25
Secbumeton	5.8	226.2	38	170.2	18	100.2	26
Siduron	6.2	233.0	38	93.8	22	137.0	16
Simetryn	5.5	214.0	44	124.0	20	95.9	26
Spinetoram	10.3	748.5	58	142.2	35	98.1	70
Spinosad A	9.7	732.6	40	142.0	30	98.1	60
Spinosad D	10.3	746.5	50	142.0	28	98.1	58
Spirodiclofen	10.5	411.1	25	71.2	10	313.0	15
Spiromesifen	10.2	371.1	15	273.1	10	255.1	25
Spirotetramat	6.9	374.2	35	330.2	15	302.2	18
Spiroxamine	6.7	298.0	40	144.0	18	100.0	30
Sulfentrazone	5.3	387.0	50	307.1	20	146.0	44
Tebuconazole	7.9	308.0	40	70.1	25	125.0	35
Tebufenozid	7.2	353.1	18	133.0	18	297.1	6
Tebufenpyrad	9.8	334.0	60	117.0	35	145.0	25
Tebuthiuron	5.6	229.0	38	172.0	18	116.0	26
Teflubenzuron	10.1	380.9	35	158.0	15	140.9	40
Temephos	10.1	467.1	35	125.0	36	418.9	18
Terbumeton	5.8	226.1	38	170.1	18	114.1	26
Terbuthylazine	6.5	231.9	31	176.0	16	134.0	25
Terbutryn	6.7	242.1	36	186.1	19	91.0	28
Tetraconazole	7.1	372.0	45	159.0	30	70.1	20
Thiabendazole	4.6	202.0	50	175.0	24	131.0	32
Thiacloprid	5.1	253.0	35	126.0	20	90.1	36
Thiamethoxam	4.1	292.0	19	211.0	13	181.0	22
Thidiazuron	5.2	221.0	25	101.9	14	93.9	15
Thiobencarb	8.4	258.2	30	125.0	23	89.0	46
Thiofanox	5.4	235.9	28	142.9	10	42.9	32
Thiophanate-methyl	5.2	343.0	25	151.0	20	311.0	12
Triadimefon	6.6	294.1	35	69.3	20	197.2	14
Triadimenol	6.6	296.1	12	227.1	15	99.1	15
Trichlorfon	4.2	257.0	26	109.0	16	79.0	26
Tricyclazole	5.3	190.0	32	163.0	20	136.0	28
Trifloxystrobin	8.9	409.0	30	186.0	20	145.0	50
Triflumizole	9.3	346.0	24	277.9	10	43.1	24
Triflumuron	8.3	359.0	28	156.1	33	139.1	16
Triticonazole	7.1	318.1	25	70.1	25	124.9	30
Vamidothion	4.6	288.0	24	146.0	11	118.0	24
Zoxamide	7.7	336.0	38	187.1	20	159.0	40

**Table A3:** GC–MS/MS parameters optimized for pesticide residues determination.

Compound Name	Retention Time (min)	Precurso Ion (m/z)	Productio Ion (m/z)	Collisio Energy (V)	Precurso Ion (m/z)	Productio Ion (m/z)	Collisio Energy (V)	Precurso Ion (m/z)	Productio Ion (m/z)	Collision Energy (V)
2,4-DDD	20.1	237.1	165.1	20	235.0	165.2	20			
2,4-DDE	18.6	248.0	176.2	30	246.1	176.2	30			
2,4’-DDT	21.2	236.8	165.1	20	235.0	165.1	20	235.0	199.0	15
4,4’-DDD	21.6	237.0	165.1	25	235.0	165.2	20	235.0	199.1	15
4,4’-DDE	19.9	318.0	248.0	20	246.0	176.1	30	317.8	246.0	20
4,4’-DDT	22.6	236.8	165.0	22	165.1	164.3	15	235.0	19.5	10
Aldrin	16.4	262.9	190.9	35	262.7	191.0	30	262.9	193.0	30
Atrazine	13.2	215.1	200.0	5	200.0	132.0	8	200.1	122.2	10
Atrazine-desethyl	12.1	172.0	69.1	15	172.0	94.1	15	172.0	104.1	15
Azinphos-ethyl	27.0	160.0	77.0	16	132.0	51.0	26	132.0	77.0	12
Benfluralin	11.9	292.1	160.1	20	276.1	202.1	15	292.1	264.0	10
Bifenox	25.2	341.1	281.0	12	172.9	137.9	16	311.0	216.0	25
Bifenthrin	23.8	181.2	165.2	25	165.1	163.6	24	181.0	179.0	12
Bromfenvinphos-methyl	17.9	294.9	79.1	30	109.0	79.0	6	294.9	109.0	16
Bromophos-ethyl	18.4	302.7	284.8	14	96.9	65.0	16	96.9	78.9	12
Bromophos-methyl	17.1	331.0	286.0	25	328.9	313.8	14	328.9	313.9	20
Bromopropylate	24.1	340.8	185.0	14	184.9	75.5	30	184.9	156.9	12
Carbophenothion	22.1	342.0	157.0	10	157.0	45.0	12	199.0	142.9	10
Chlorbufam	13.2	127.0	65.0	35	53.1	27.1	10	127.0	100.0	15
Chlordene	14.0	66.1	39.1	20	66.1	40.1	15	66.1	65.1	10
Chlorfenapyr	20.6	248.9	112.0	24	59.1	29.1	10	59.1	31.1	5
Chlorfenprop-methyl	11.4	196.0	165.1	10	165.0	102.0	18	165.0	137.0	10
Chlorfenson	19.7	177.0	113.1	10	111.0	75.1	15	175.0	111.0	10
Chlorfenvinphos	17.8	323.0	266.9	14	266.9	159.0	16	266.9	203.0	10
Chlorobenzilate	20.8	139.0	74.9	26	111.0	75.1	14	139.0	111.0	12
Chlorothalonil	15.6	265.8	133.0	36	228.8	168.0	8	265.8	170.0	24
Chlorpropham	12.0	213.0	127.0	14	171.0	127.0	8	213.0	171.0	6
Chlorpyrifos-methyl	15.1	285.9	93.0	20	125.0	47.0	12	125.0	79.0	6
Chlordane cis	19.1	376.6	268.0	20	372.9	266.1	20	374.9	265.8	20
Chlordane trans	18.9	374.7	265.9	22	271.7	236.8	12	373.0	264.1	20
Cyanazine	16.8	198.0	55.1	24	198.0	91.0	10	198.0	157.0	8
Cyanophenphos	22.3	169.0	77.1	22	157.0	77.1	22	169.0	141.0	8
Cyanophos	13.8	243.0	109.0	10	125.0	79.0	60	125.0	96.9	6
Cyflutrin beta	29.0	163.0	65.1	26	163.0	91.1	12	163.0	127.1	6
Cyflutrin gama	29.1	206.0	151.1	18	163.0	91.1	12	163.0	127.0	6
Cyhalofop butyl	26.1	256.0	91.1	24	256.0	120.0	10	256.0	157.8	30
Cyhalothrin gamma	25.9	208.1	151.8	28	181.0	151.9	22	208.1	180.9	8
Cyhalothrin lambda	25.5	208.1	180.9	8	180.9	151.9	22	197.0	141.1	10
Cypermethrin ion 1	29.3	180.9	152.1	20	163.0	91.1	12	163.0	127.1	6
Cypermethrin ion 2	29.5	180.9	151.9	18	163.0	91.1	12	163.0	127.0	6
Cypermethrin ion 3	29.6	163.0	91.0	12	163.0	127.0	6	163.0	152.1	12
Cypermethrin ion 4	29.7	180.9	152.2	20	163.0	91.1	12	163.0	127.1	6
Demeton O	11.4	171.1	115.0	10	88.1	59.8	6	89.1	61.0	8
Demeton S	12.8	170.0	114.0	8	114.0	81.0	14	142.5	114.9	6
Diallate	12.4	234.1	150.0	20	86.1	43.1	5	234.1	192.1	10
Diazinon	13.4	179.1	121.5	26	137.1	54.1	20	137.1	84.1	12
Dichlorvos	8.4	186.9	93.0	12	109.0	79.0	6	185.0	93.0	12
Diclofop-methyl	23.0	340.0	253.0	10	252.9	126.9	36	253.0	162.1	15
Dicloran	13.5	205.9	147.9	20	175.9	148.0	10	205.9	176.0	10
Dicofol	17.2	250.0	139.0	10	111.0	75.0	15	139.0	111.0	15
Dieldrin	20.0	277.0	240.0	8	262.7	193.0	39	262.9	191.0	38
Dinitramine	13.9	260.7	194.7	18	215.9	196.0	8	260.7	241.0	8
Dioxabenzofos	12.4	216.0	138.0	8	183.0	153.0	8	216.0	201.0	8
Diphenylamine	11.9	167.1	139.4	26	167.1	140.1	18	167.1	166.1	16
Disulfoton	13.9	185.9	96.9	16	88.0	45.0	18	88.0	59.8	6
Endosulfan alpha	19.2	240.9	170.0	20	194.7	125.0	22	194.7	159.4	8
Endosulfan beta	21.8	240.9	205.9	15	158.9	123.0	12	194.7	159.0	8
Endosulfan sulfate	23.1	271.8	237.0	10	238.9	204.0	15	271.7	235.0	12
Endrin	20.8	280.0	245.0	8	245.0	173.0	22	262.0	192.0	30
Endrin Aldehyde	22.2	344.9	281.0	8	173.0	138.1	16	249.8	214.9	24
EPTC	9.3	189.1	43.1	16	128.1	43.1	10	189.1	128.1	6
Esfenvalerate	31.3	181.1	152.1	20	125.0	89.0	22	167.0	125.0	12
Ethalfluralin	11.8	315.9	276.1	8	276.0	202.0	14	276.0	248.1	8
Ethion	20.9	230.9	128.9	22	153.0	97.0	10	230.9	174.9	12
Ethoprophos	11.7	200.0	158.0	6	157.9	96.9	16	157.9	113.9	6
Fenamiphos	19.2	303.1	195.2	8	154.0	139.0	10	216.9	202.0	12
Fenchlorphos	15.4	287.0	272.0	20	124.9	79.0	6	169.0	110.4	6
Fenitrothion	16.0	277.0	109.0	16	125.0	79.0	8	277.0	260.0	6
Fenpropathrin	24.3	181.0	126.8	28	97.1	55.1	6	181.0	151.9	22
Fensulfothion	21.1	307.9	96.9	26	293.0	97.0	16	293.0	125.0	10
Fenthion	16.5	278.0	109.0	18	245.3	125.0	12	278.0	169.0	14
Fenvalerate	31.0	225.0	119.0	18	125.0	89.0	22	167.0	125.0	12
Flucythrinate cis	29.8	451.0	199.0	10	157.0	107.0	15	199.0	157.0	10
Flucythrinate trans	30.1	451.0	199.0	10	157.0	107.0	15	199.0	157.0	10
Folpet	18.7	262.0	130.0	15	104.0	76.0	10	130.0	102.0	12
Formothion	14.8	170.0	93.0	8	125.0	79.0	8	126.1	93.0	6
HCH alpha	12.9	218.9	182.9	8	216.9	180.9	8	216.9	180.9	8
HCH beta	13.8	218.9	182.9	8	109.0	49.0	25	182.9	147.0	10
HCH delta	15.3	218.9	182.9	10	181.0	145.0	15			
HCH gamma (Lindane)	14.6	218.9	182.9	8	180.9	145.0	10	216.9	180.9	8
Heptachlor	15.4	273.8	238.8	15	100.0	65.1	10	271.8	236.9	15
Heptachlor epoxide	17.8	354.9	264.9	10	81.1	53.1	10	352.9	262.9	10
Heptenophos	11.0	215.0	200.0	8	124.0	62.9	28	124.0	89.0	12
Hexachlorobenzene	13.1	285.8	213.9	30	283.8	213.9	30	283.8	248.9	20
Isodrin	17.4	194.9	159.0	20	66.1	65.1	10	192.9	123.0	30
Isofenphos	17.5	213.0	121.0	14	185.0	121.0	10	213.0	185.0	6
Malaoxon	14.9	127.0	99.0	6	99.0	71.0	8	127.0	109.0	12
Malathion	15.9	173.1	99.0	12	92.8	63.0	8	125.0	79.0	8
Mefenpyr-diethyl	23.4	299.0	253.1	10	253.0	127.7	36	253.0	189.3	22
Methacrifos	10.3	240.0	180.0	10	125.0	79.0	8	180.0	93.0	10
Methidathion	18.7	302.6	284.9	14	145.0	58.0	14	145.0	85.0	6
Methoxychlor	24.4	227.1	141.1	35	227.0	184.0	20	227.1	169.1	25
Mirex	26.1	273.8	238.9	15	236.8	142.9	20	271.8	236.9	15
Nitrofen	21.1	285.0	164.0	20	202.0	139.0	21	283.0	162.1	20
Oxychlordane	17.8	388.8	262.9	15	184.9	84.9	26	184.9	121.0	12
Oxyfluorfen	20.1	300.0	167.0	25	252.0	196.1	20	300.0	223.0	20
Paraoxon-ethyl	15.6	149.0	91.1	10	109.0	81.0	10	149.0	102.0	16
Parathion-ethyl	16.8	291.0	109.0	12	109.0	81.0	10	124.9	97.0	6
Parathion-methyl	15.4	263.0	109.0	12	124.9	47.0	12	124.9	79.0	6
Pentachloroaniline	15.0	264.8	193.9	20	262.9	191.9	20	264.8	229.9	10
Pentachlorobenzene	11.0	249.9	214.9	15	247.9	142.0	40	247.9	212.9	15
Pentanochlor	16.1	143.1	106.1	25	71.1	43.1	5	141.0	77.1	30
Permethrin cis	27.4	183.1	153.0	12	163.0	91.1	12	183.1	168.0	12
Permethrin trans	27.7	183.0	153.0	14	183.0	165.1	10	183.0	168.1	10
Phorate	12.4	260.0	75.0	8	75.0	47.0	8	121.0	65.0	8
Phosalone	26.0	182.0	74.8	30	121.1	65.0	10	182.0	111.0	14
Phosphamidon	14.6	264.1	127.0	12	127.0	94.9	16	127.0	109.0	12
Pirimiphos-ethyl	16.7	318.1	166.1	12	304.0	168.1	12	318.1	182.1	10
Pirimiphos-methyl	15.5	305.1	180.1	8	290.1	125.0	20	290.1	233.0	8
Procymidone	18.2	283.0	96.1	10	96.0	53.0	16	96.1	67.1	10
Propanil	15.3	219.0	163.0	10	160.9	125.7	16	161.0	99.0	25
Prothiofos	19.3	308.9	239.0	14	266.7	220.9	18	266.7	238.9	8
Pyrazophos	26.6	231.9	204.1	10	221.0	148.7	14	221.0	193.1	8
Quinalphos	17.9	157.1	102.0	22	146.0	118.1	10	157.1	129.0	14
Quintozene	13.8	248.8	213.9	15	236.8	119.0	25	236.8	142.9	25
Resmethrin	22.9	143.1	128.1	10	123.1	81.1	10	128.1	127.1	20
Simazine	13.2	186.0	91.0	8	172.7	138.0	6	172.7	172.2	8
Sulprofos	21.6	322.0	156.1	10	156.0	108.0	30	156.0	141.0	14
Tetrachlorvinphos	18.8	330.8	109.0	18	109.0	79.0	6	328.9	109.0	18
Thiometon	12.8	125.0	47.0	14	88.0	59.8	6	125.0	79.0	8
Tolclofos-methyl	15.3	266.8	252.0	12	265.0	219.9	20	265.0	250.0	12
Triallate	13.9	268.0	183.9	18	86.1	43.3	6	268.0	226.0	12
Trichloronate	16.9	297.0	269.0	12	268.9	222.9	20	270.8	224.9	22
Trifluralin	11.8	306.1	159.7	20	306.1	206.0	10	306.1	264.1	8
Vinclozolin	15.0	286.9	214.0	15	241.1	58.1	12	241.1	184.1	10

## Appendix B

**Table A4:** Average recovery, relative standard deviation (RSD), limit of detection (LOD), and limit of quantification (LOQ) in representative cereal (wheat) for mycotoxins.

Compound Name	Average Recovery %	Repeatability RSD%	LOD µg/kg	LOQ µg/kg
Aflatoxin B1	99.1	5.4	0.30	1.0
Aflatoxin B2	101.2	13.9	0.15	0.5
Aflatoxin G1	104.9	7.8	0.30	1.0
Aflatoxin G2	96.9	5.8	0.15	0.5
Deoxynivalenol	111.1	4.8	61	200
Fumonisin B1	101.4	3.6	46	150
Fumonisin B2	100.4	3.3	46	150
Zearalenone	97.2	4.0	9	30
T-2 toxin	97.3	7.4	3	10
HT-2 toxin	93.5	13.8	3	10
Ochratoxin A	94.3	6.0	0.30	1.0

**Table A5:** Average recovery, RSD, LODs, and LOQs in representative cereal (wheat) for found pesticide residues.

Compound Name	Average Recovery %	Repeatability RSD%	LOD µg/kg	LOQ µg/kg
Piperonyl butoxide	89.8	14.8	0.003	0.010
Chlorpyriphos	70.6	6.8	0.003	0.010
Epoxiconazole	94.8	7.4	0.003	0.010
Thiofanox	82.8	9.8	0.003	0.010
Tebuconazole	93.0	9,8	0.003	0.010
Thiametoxam	72.8	6.5	0.003	0.010
Carboxin	83.1	19.2	0.003	0.010
Cypermethrin I	80.7	11.4	0.003	0.010
Cypermethrin II	87.6	19.0		
Cypermethrin III	72.2	15.2		
Cypermethrin IV	82.4	5.3		
Pyrimiphos-methyl	86.3	14.8	0.003	0.010

**Table A6:** Average recovery, RSD, LODs, and LOQs in representative cereal (wheat) for heavy metals.

Analyte	Average Recovery %	Repeatability RSD%	LOD µg/kg	LOQ µg/kg
Lead	103.8	1.87	0.008	0.025
Cadmium	90.1	3.88	0.002	0.006
Arsenic	97.8	2.77	0.0003	0.01
Mercury	98.8	4.84	0.0003	0.001

**Table A7:** Proficiency testing results for cereals.

Proficiency Testing Scheme	Matrix	Analyte	z-Score
Romer Labs CSSMY014–M18161DZ	Wheat	Deoxynivalenol	0.3
		Zearalenone	1.6
Bipea 03-0531	Barley	Aflatoxin B1	−1.48
		Aflatoxin B2	0
		Aflatoxin G1	−0.08
		Aflatoxin G2	0.35
		T-2 toxin	−0.21
		HT-2 toxin	0.15
Romer Labs CSSMY017-M19411AF	Maize	Aflatoxin B1	−1.7
		Aflatoxin B2	−1
		Aflatoxins sum	−1.6
		Fumonisin B1	−0.4
		Fumonisin B2	−0.4
		Fumonisin sum	−0.7
Bipea 19b-342	Maize	Azoxystrobin	1.38
		Boscalid	0.4
		Piperonyl butoxide	0.36
		Metalaxyl	1.85
		Propiconazole	0.73
		Pirimicarb	0.6
EUPT-CF11	Oat flour	Azoxystrobin	−0.6
		Carbendazim	2.1
		Chlordane trans	0.3
		Chlorpyrifos	−0.7
		Cyhalothrin lambda	−1.8
		Cyproconazole	−3.8
		Cyprodinil	−0.8
		Endosulfan alpha	0.9
		Fenbuconazole	0.7
		Fenpropimorph	−0.3
		Fludioxonil	−0.2
		Flusilazole	0.7
		Flutolanil	−1
		Metconazole	0.9
		Prothiofos	0.0
		Pyraclostrobin	0.1
		Tebuconazole	0.3
Bipea 32a-90	Wheat	Cadmium	−1.18
		Lead	−1.07
Fapas 07307	Rice Cakes	Arsenic	0.0
		Cadmium	0.0
		Lead	1.6
